# Observational study of compliance with infection control practices among healthcare workers in subsidized and private residential care homes

**DOI:** 10.1186/s12879-021-05767-8

**Published:** 2021-01-14

**Authors:** Jessie Kit Ling Au, Lorna Kwai Ping Suen, Simon Ching Lam

**Affiliations:** 1 School of Nursing, The Hong Kong Polytechnic University, Hong Kong, Hong Kong SAR; 2 School of Nursing, Tung Wah College, Hong Kong, Hong Kong SAR; 3 Squina International Centre for Infection Control, The Hong Kong Polytechnic University, Hong Kong, Hong Kong SAR

**Keywords:** Residential care homes, Infection control practice, Healthcare workers, Hand hygiene, Use of gloves, Respiratory protection

## Abstract

**Background:**

The elderly population in Hong Kong is rapidly growing, and the need for residential care homes (RCHs) is increasing. The risk of being infected with micro-organisms increases among the frail and the vulnerable elderly population as their immunity system begins to deteriorate. Furthermore, the residents in RCHs are at high risk of healthcare-associated infections (HAIs) due to the confined living environments and individual co-morbidities. In relation to this, infection control practice (ICP) is considered a crucial and effective approach in preventing HAIs. This study aimed to observe the daily ICP of healthcare workers in RCH settings.

**Methods:**

An observational study was conducted to observe daily ICP among healthcare workers in private and subsidized RCHs. Each RCH was separated into different units based on the location (common area and bedroom area) and nature of residents for successive days. The ICP episodes were observed until 200 opportunities in each unit. The ICP episodes were recorded by an electronic tool called “eRub,” which is an ICP checklist based on international guidelines.

**Results:**

The most frequent observed ICP episodes were hand hygiene (*n* = 1053), the use of gloves (*n* = 1053) and respiratory protection (*n* = 1053). The overall compliance of hand hygiene was poor, with only 15% of participants performing this during the “five moments for hand hygiene.” Furthermore, the observations showed that 77.9% improperly performed the use of gloves, and 31.8% failed to wear a mask during the care provision for the elderly. However, the results showed that most healthcare workers can wear the mask in a proper way when they should. Generally, the personal care workers were the worst in terms of hand hygiene and use of gloves compared with the other types of healthcare workers.

**Conclusions:**

Despite the fact that the practice of hand hygiene, the use of gloves, and respiratory protection were the important elements of ICP, overall compliance to these elements was still poor. Personal care workers had the most frequent contact with the residents, but they had the worst compliance rate. Hence, continued monitoring and training among healthcare workers is needed, particularly personal care workers, in this healthcare service setting.

## Background

Healthcare-associated infections (HAIs) are the infections incurred by patients during the process of caring in a healthcare setting. In 2019, The World Health Organization reported that about 7 and 10% of the populations in developed and developing countries, respectively, suffer from HAIs. It is a widespread issue that has threatened the patients’ safety within healthcare delivery systems around the world [1–3].

It has been estimated that 1.13–2.68 million infections occurred in nursing homes in the US in 2013 [4]. A study of HAIs in a long-term care facility (HALT) project in a Dutch facility from 2010 to 2017 showed that the average infection prevalence rates were 6.7 and 2.2% from 2007 to 2011 and from 2012 to 2017, respectively [5]. Another similar study estimated that 2.6 million residents were confirmed to have HAIs each year in Europe [6].

In Hong Kong, the overall prevalence of infection in RCHs was 2.7% in 2016, which was lower than that in 2006 (5.7%) [7]. Methicillin-resistant *Staphylococcus aureus* (MRSA), a common pathogen causing HAIs, is endemic in Hong Kong. The prevalence of MRSA was estimated at 30.1% in Hong Kong RCHs, much higher than those reported in previous studies in 2005 (2.8%) and 2001 (21.6%) [8, 9]. The MRSA transmission was also reported to be more serious in RCHs than in hospitals [10].

Most of HAIs can be prevented effectively through the optimum infection control practice (ICP) [11]. ICP aims to prevent the risk of disease transmission by contact with blood, bodily fluid, mucous membranes, and non-intact skin. This implies that compliance with ICP is closely related to the risk of HAIs, yet empirical studies have shown that ICP compliance is suboptimal among healthcare practitioners in different countries [1, 12, 13]. However, most of the ICP compliance studies have focused on the hospital setting. To date, studies on ICP compliance among healthcare workers in RCHs in Hong Kong are limited, and the ICP has yet to be fully documented in this setting.

RCHs are a kind of long-term care facility that provide different levels of care to the elderly with social and/or physical problems. Long-term care facilities are mainly classified into subsidized and private institutions. All RCHs must operate in accordance with the code of practice licensed by the Residential Care Homes Ordinance [14]. The code of practice includes infection control, which requires RCHs to follow the guidelines developed by the Centre for Health Protection of the Department of Health. Therefore, healthcare workers should adopt ICP while performing patient care in RCHs.

Meanwhile, the population of Hong Kong is rapidly aging. According to the Hong Kong Census and Statistics Department, the population over 65 is estimated to increase from 17% in 2016 to 37% in 2066. Meanwhile, epidemiologic studies mentioned that the elderly tend to have worse complications if they sustain infections. The frail and vulnerable elderly populations in RCHs are easily infected with micro-organisms. Moreover, the residents in RCHs are at high risk of HAIs because of the confined living environments and individual co-morbidities [7]. In the RCHs, the residents are living collectively, which means they share many facilities and common areas with others [15]. Such a living situation within a confined environment poses a risk of cross infection, particularly those transmissions through air-borne particles, water droplets, and personal contact. In addition, because of the demand for basic care, many frail residents must always be in close contact with healthcare workers. This increases a risk of HAI transmission between the staff and residents [16]. Finally, most of the residents in RCHs are suffering from multiple diseases, e.g., Diabetes Mellitus, stroke, heart disease, etc. [17, 18], which are likely to cause HAIs among the elderly. These are the leading causes of morbidity and mortality among the elderly and are known to increase burden on the healthcare system. Therefore, compliance with ICP among healthcare workers is important and has drawn researchers’ attention in the past 20 years. As such, the current study aimed to observe the ICP compliance of healthcare workers in private and government-subsidized RCHs.

## Methods

### Design and methods

We collected the demographic data during the first on-site visit in each RCH unit included in this study. The demographic data included the following: number of residents, number of healthcare workers, availability of alcohol-based hand rubs (ABHR), and availability of washing facilities. The observations of ICP include hand hygiene, use of gloves, use of personal protective equipment (PPE), respiratory hygiene, handling of sharp equipment, decontamination of equipment, waste management, and environmental cleaning [13, 14, 19].

We conducted an observational study to observe ICP among healthcare workers in private and government-subsidized RCHs. In this study, the researcher (as a non-participant observer) was a qualified registered nurse who had been trained and had accumulated experience in patient care. This ensured that the researcher was familiar with ICP. The researcher recorded observations at any time in different shifts (morning or afternoon shifts) every day (from Monday to Sunday) in different units of both the private and subsidized RCHs. This allowed for the collection of comprehensive data on staff behavioral changes in different shifts during the weekdays and weekends [19]. For such consecutive observations done for 2–3 weeks in each RCH, the Hawthorne effect was reduced by desensitizing the healthcare workers to the frequent presence of the observer. Such a method has been suggested elsewhere [20] and is considered more reliable for continuous sampling. The observed healthcare workers were chosen randomly to minimize the selection bias. Once the observed staff was chosen, the researcher did not interrupt the care procedure. As the opportunities occurred, the performances were recorded by using an electronic tool, called the “eRub.” According to the guideline, hand hygiene involved ABHR and the use of soap and water for at least 20 s under the condition of the “five moments for hand hygiene” [21].

### Setting

The studied setting included both subsidized and private RCHs with similar sizes in order to reduce the confounding factors in different RCHs. A list of RCHs was retrieved from the Social Welfare Department to identify the private and subsidized care attention homes for the elderly. Such homes for the elderly are the most common types of RCHs in Hong Kong, making up 80% of the total. Thus, this can be considered as a representative sample. Medium-sized RCHs with three floors and around 200 residents were invited by contacting the superintendent/managers by phone.

The subsidized RCHs had 180–220 beds, 99% of which were occupied throughout the year. Single rooms were unavailable and four to eight residents of the same gender shared a partitioned room per bed unit. The toilet and bathroom were shared within a room. The RCHs employed over 90 staff members (staff-to-resident ratio is about 1:2-3) to provide diverse care and services. Staff in RCHs included professional staff (i.e., registered nurses, enrolled nurses, physiotherapist and occupational therapist), health workers, personal care workers, and workmen.

In comparison, in private RCHs, specific levels or categories of care do not exist. These homes accommodate about 150–300 residents who require diverse levels of care, ranging from minimal personal care to medium nursing care. Only about 70–80% of beds are occupied throughout the year. Some single rooms are provided, but rooms shared by 4–12 residents of the same gender are common. However, toilets and bathrooms are shared with all residents in the same floor. The common areas include the combined sitting and dining room, the recreation room, and the consultation and treatment rooms. In terms of staffing, these homes employ about 50–70 staff members (approximate ratio of staff to residents is 1:4-5) to provide a diverse range of care and services, including basic care, nursing care, social and support services, food preparation, and housekeeping. The core staff includes professional staff (i.e., registered nurses and enrolled nurses), personal care workers and workmen. Social workers, physiotherapists and occupational therapists work on part-time basis or are shared within and among the organizations. Although the duties and work patterns are similar to those of subsidized RCHs, the ratios of different staff grades vary. For example, such homes have scarce nursing staff but more personal care workers. According to the regional authority body and statute [14], annual infection control training for all healthcare workers working in RCH is mandatory.

For observations, in every RCH, we separated each floor into several units depending on the geographic location (i.e., common area and bedroom area), because of the difference in intensity and types of care provided. A common area (e.g., dining area) is the place shared by all the residents, and most of the group activities are held there. Bedrooms are for resting and receiving direct personal care, such as wound dressing and tube feeding. Finally, there are 6 units in the subsidized RCHs (3 units of common areas, 3 units of residential bedrooms) and 5 units in private RCHs (2 units common of areas and 3 units of residential bedrooms). The floor with only bed-bound residents (18 residents) in private RCHs was not accessible for making observations, because the relatives of the residents refused to allow the observations around.

### Samples and sampling methods

According to the World Health Organization (WHO) guideline [21], the minimum sample size for hand hygiene audit is 200 opportunities per unit per observation period, and each observation session should be 20 min (up to 10 min longer or shorter) with no more than three observed participants to be observed simultaneously. Our researcher strictly followed these guidelines. The observation targets were healthcare workers, including nurses (registered nurses and enrolled nurses), allied healthcare professionals (AHCPs, i.e., physiotherapists and occupational therapists), health workers (HWs) and personal care worker (PCWs), who were the ones directly giving care for the elderly.

### Measurement

We recorded the ICP episodes by using an electronic tool called “eRub,” which is the checklist of ICP by international guideline [14, 21]. The observation items in the eRub include hand hygiene performance, use of gloves, respiratory hygiene, disinfecting used surfaces/equipment, handling of linen, handling of clinical waste, handling of sharp equipment, use of PPEs, and likelihood of hand colonization. This electronic tool is convenient for observers as it allows them to gather data immediately and saves time for data entry. Furthermore, using the mobile phone for data collection is better than the pen-and-paper method, because it can reduce the errors committed in gathering large amounts of data and can be more unobtrusive in performing observation.

The interrater agreement test between a research nurse (one observer for all data collection) and infection control expert was established by using WHO Training Film in the Implementation Toolkit. The score of > 0.8 reliability was achieved before the commencement of data collection. This test is important in ensuring the consistency of an observer’s observational rating and increasing the validity of the data obtained [22].

A three-point scale was used to calculate the hand hygiene performance score (0 = missing to perform, 1 = performed with hand hygiene < 20 s, and 2 = well performed with hand hygiene > 20 s), use of gloves (0 = did not perform, 1 = improperly performed, and 2 = properly performed), and respiratory hygiene (0 = did not perform, 1 = improperly performed, and 2 = properly performed).

### Data analysis

Descriptive and inferential statistics were used to analyze the data by using SPSS (Window version 25.0). The ICP episodes were summarized by descriptive statistics. The significant differences in performance scores between healthcare workers working in private and subsidized RCHs and between those in common areas and residential room areas were determined by independent t-tests. The different performance scores of the healthcare workers were compared by using One-way ANOVA test and post-hoc analyses. Two variables (hand hygiene performance and use of gloves) were calculated by Pearson product moment correlations.

## Results

**Fig. 1:**
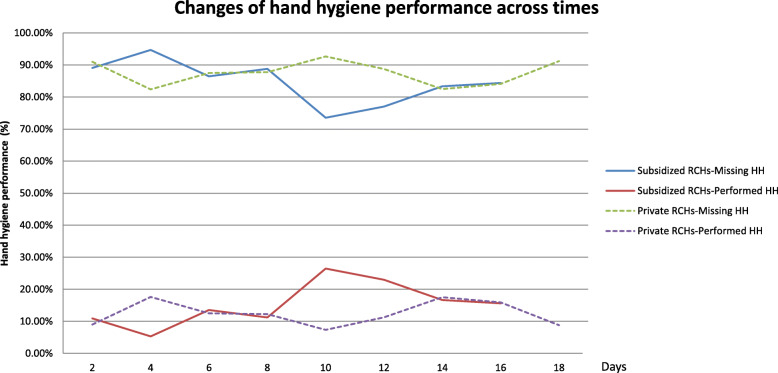
Changes of hand hygiene (HH) performance across times

The demographic data included the number of residents, the number of healthcare workers, number of sinks for handwashing, and number of ABHRs in private and subsidized RCHs. The data are presented in Table 1. The final sample contained 1053 (46.1%) and 1231 (53.9%) observations in private and subsidized RCHs, respectively. Hand hygiene performance, use of gloves, and respiratory protection were the most observed episodes in ICP. Other episodes of ICP, such as disinfecting used surfaces/equipment, handling of linen, handling of clinical waste, handling of sharp equipment, use of PPEs, and the likelihood of hand colonization were uncommon and difficult to record in this setting. The sampled episodes of healthcare workers included PCWs: 1474 (64.5%), HW: 349 (64.5%), nurses: 349 (18.4%), AHCPs: 36 (1.6%), and doctors: 5 (0.2%). The overall change of hand hygiene performance was minimal and steady over the observation period in both types of RCHs (Fig. 1).

**Table 1:** Demographic data of RCHs

Demographics	Total n (%)	Subsidized RCHs n (%)	Private RCHs n (%)
Number of observations	2284 (100)	1231 (53.9)	1053 (46.1)
Number of residents	461 (100)	212 (45.9)	249 (54.1)
Number of healthcare workers	140 (100)	78 (55.7%)	62 (55.7)
- Nurses^a^:	31 (22.1)	15 (19.2)	16 (25.8)
- Health workers:	17 (12.1)	9 (11.5)	8 (12.9)
- Personal Care workers:	92 (65.7)	54 (69.2)	38 (61.2)
Number of sinks for hand washing	157 (100)	82 (52.2)	75 (47.8)
Number of alcohol hand rubs	50 (100)	15 (30)	35 (70)

^a^ include registered nurses and enrolled nurses

The most frequently observed ICP elements were hand hygiene, use of gloves, and respiratory practice. In this setting, other elements of ICP were seldom observed. Thus, our analysis focused on hand hygiene, use of gloves and respiratory hygiene score among healthcare workers in subsidized and private RCHs. Episodes of ICP observations among AHCPs and doctors were also seldom observed in this setting. Thus, nurses, doctors, and AHCPs were grouped as professional staff categories for further analysis (Table 2).

**Table 2:** Comparison between subsidized and private RCHs regarding hand hygiene, use of gloves and respiratory protection performance of different healthcare workers

	Subsidized RCH			Private RCH		
	PCWs, n (%)	HWs, n (%)	Professional staff^a^, n (%)	PCWs, n (%)	HWs, n (%)	Professional staff^a^, n(%)
**Hand Hygiene**						
Missing	742 (87.6)	185 (80.8)	99 (63.9)	553 (88.2)	112 (93.3)	251 (82)
Performed	98 (11.6)	38 (16.6)	47 (30.3)	66 (10.5)	8 (6.7)	37 (12.1)
Well performed	7 (0.8)	6 (2.6)	9 (5.8)	8 (1.3)	0 (0.0)	18 (5.9)
**Use of gloves**						
Not applicable	584 (68.9)	206 (90)	134 (86.5)	330 (52.6)	53 (44.2)	263 (85.9)
Missing	3 (0.4)	3 (1.3)	1 (0.6)	5 (0.8)	0 (0.0)	1 (0.3)
Improperly performed	219 (25.9)	11 (4.8)	5 (3.2)	239 (38.1)	60 (50)	22 (7.2)
Properly performed	41 (4.8)	9 (3.9)	15 (9.7)	53 (8.5)	7 (5.8)	20 (6.5)
**Respiratory protection**						
Missing	214 (25.3)	71 (31.0)	51 (32.9)	273 (43.5)	3 (2.5)	114 (37.3)
Improperly performed	8 (0.9)	3 (1.3)	2 (1.3)	1 (0.2)	0 (0.0)	0 (0.0)
Properly performed	625 (73.8)	155 (67.7)	102 (65.8)	353 (56.3)	117 (97.5)	192 (62.7)

*n* = total numbers of observed infection control practice episodes

% = percentage of compliance in each of the total number of observed infection control practice episodes

^a^Professional staff include nurses, doctors, and allied health professionals

### Hand hygiene performance

Healthcare workers should perform hand hygiene during the “five moments for hand hygiene.” In this study, the observation numbers of five moments showed 30.9% before touching a resident; 1.4% before a clean or aseptic procedure (e.g., before nasogastric tube feeding or changing dressing); 1.4% after blood, body fluid, secretion, excreta, wound, or mucous membrane exposure risk (e.g., after changing diaper); 50.2% after touching a resident; and 16.2% after touching contaminated items or the residents’ surrounding environment.

The HWs had poor hand hygiene performance before patient contact (98% failed to perform) and after coming into contact with the patients’ surrounding (97% failed to perform). In comparison, they showed better hand hygiene after body fluid exposure risk (only 54% failed to perform).

The hand hygiene performance of the professional staff and HWs is better than that of PCWs (Table 2). The best and worst hand hygiene practices were observed among the professional staff and the PCWs, respectively. The professional staff (well-performed: 5.8%, performed: 30.3%) and HWs (well-performed: 2.6%, performed: 16.6%) in subsidized RCHs performed better than the professional staff (well-performed: 5.9%, performed: 12.1%) and HWs (well performed: 0%, performed: 6.7%) in private homes (Table 2). Post-hoc analysis using the Bonferroni multiple comparison also indicated that the hand hygiene of professional staff was significantly different from those of HWs and PCWs (F = 27.54, *p* < 0.001) (Table 3), wherein the professional staff performed better than HWs and PCWs.

**Table 3:** Compliance with infection control guidelines among healthcare workers (HCWs)

	Professional staff ^a^, n (%)	HWs, n (%)	PCWs, n (%)	ANOVA test, ***p***-value
**Hand hygiene**				F = 27.54, *p* < 0.001
Missing	350 (75.92)	297 (85.1)	1295 (87.86)	
Performed	84 (18.22)	46 (13.18)	164 (11.13)	
Well performed	27 (5.86)	6 (1.72)	15 (1.02)	
**Use of gloves**				F = 40.13, *p* < 0.001
Missing	2 (3.13)	3 (3.33)	8 (1.43)	
Improperly performed	27 (42.19)	71 (78.89)	458 (81.79)	
Properly performed	35 (54.69)	16 (17.78)	94 (16.79)	
**Respiratory protection**				F = 11.08, *p* < 0.001
Missing	165 (35.79)	74 (21.2)	487 (33.04)	
Improperly performed	2 (0.43)	3 (0.86)	9 (0.61)	
Properly performed	294 (63.77)	272 (77.94)	978 (66.35)	

*n* = total numbers of observed infection control practice episodes

% = percentage of compliance in each of the total number of observed infection control practice episodes

^a^Professional staff include nurses, doctors, and allied health professionals

Overall hand hygiene performance among healthcare workers was inadequate in RCHs. The missing rate in private RCHs (87%) was slightly higher than that in subsidized RCHs (83%) (Table 4). The use of soap and water to wash hands (82%) was obviously higher than using ABHR (18%) among healthcare workers. However, the use of ABHR was more frequent among the professional staff (8%) than HWs (4%) and PCWs (1%) (Table 5). T-test results showed there was no significant difference in hand hygiene performance between private and subsidized RCHs (t = 1.65, *p* = 0.1) and between common areas and bedroom areas (t = 0.74, *p* = 0.92) (Table 4).

**Table 4:** Compliance with infection control guidelines in type of RCHs (subsidized and private RCHs) and location (common area and bedroom area)

	Overall, n(%)	Subsidized RCHs, n(%)	Private RCHs, n(%)	t-test, ***p*** value	Common areas, n(%)	Bedroom areas, n(%)	t-test, ***p*** value
**Hand hygiene**				t = 1.65, *p* = 0.10			t = 0.74, *p* = 0.92
Missing	1942 (85.03)	1026 (83.35)	916 (86.99)		881 (84.87)	1061 (85.5)	
Performed	294 (12.87)	183 (4.87)	111 (10.54)		136 (13.1)	158 (12.68)	
Well performed	48 (2.10)	22 (1.79)	26 (2.47)		21 (2.02)	27 (2.70)	
**Use of gloves**				t = −6.81, *p* < 0.001			t = −3.84, *p* < 0.001
Missing	13 (1.82)	6 (1.95)	5 (1.23)		3 (1.05)	10 (2.34)	
Improperly performed	556 (77.87)	235 (76.55)	321 (78.87)		240 (83.62)	316 (74.00)	
Properly performed	145 (20.31)	66 (21.5)	81 (19.9)		44 (15.33)	101 (23.65)	
**Respiratory protection**				t = 4.74, *p* < 0.001			t = −1.841.84, *p* = 0.06
Missing	726 (31.79)	338 (27.46)	390 (37.04)		351 (33.82)	375 (30.1)	
Improperly performed	14 (0.61)	13 (1.06)	1 (0.09)		5 (0.48)	9 (0.72)	
Properly performed	1544 (67.6)	880 (71.49)	662 (62.87)		682 (65.7)	862 (69.8)	

*n* = total numbers of observed infection control practice episodes

% = percentage of compliance in each of the total number of observed infection control practice episodes

**Table 5:** Use of handrub and handwashing among healthcare workers

	Occupations		
	PCWs, n (%)	HWs, n (%)	Professional staff, n (%)
**Alcohol-based handrub**	11 (1)	14 (4)	38 (8)
**Handwashing using soap and water**	168 (11)	38 (11)	73 (16)
**Hand hygiene missing**	1295 (88)	297 (85)	350 (76)

### Use of gloves

There was a low number of observations before aseptic task (1.4%) and after body fluid exposure risk (1.4%) moments. About 67% were presented as not applicable among the use of gloves observations. However, the observations showed that 77.9% improperly performed this practice (Table 3). For example, most of the healthcare workers did not even change gloves between patient contacts.

The observations for the proper use of gloves were 54.7% in professional staff (mainly nurses), 17.8% in HWs, and 16.8% in PCWs. A significant difference was found in the performance score of using gloves via the ANOVA test (F = 40.13, *p* < 0.001) (Table 3). Post-hoc analyses showed that the professional staff performed significantly better than PCWs (*p* < 0.001) and HWs (*p* < 0.01) (Table 3).

On the one hand, there was a slightly higher frequency of improper use of gloves in private (79%) than in subsidized (77%) RCHs (Table 4). On the other hand, there were 6 and 5% who failed to use gloves in subsidized and private RCHs, respectively. A significant difference in the proper use of gloves between subsidized and private RCHs (t = − 6.81, *p* < 0.001) and between common areas and bedroom areas (t = − 3.84, *p* < 0.001) was found (Table 4).

### Respiratory protection

Around 31.8% failed to wear the mask as needed (Table 4). However, most of them knew how to wear the required mask properly. Moreover, HWs performed better respiratory protection (77% performed properly) than the professional staff (65%) and PCWs (66%). A significant difference in respiratory protection among occupations was found (F = 11.08, *p* < 0.001) (Table 3).

The practice of respiratory protection was better in subsidized (72%) than in private RCHs (63%). A significant difference was found in the practice of respiratory protection between subsidized and private RCHs (t = 4.74, *p* < 0.001). However, there was no significant difference of such a practice between common areas and bedroom areas (t = − 1.84, *p* = 0.06) (Table 4).

### Association between hand hygiene performance and use of gloves

There was a negative correlation between the proper use of gloves and hand hygiene performance (r = − 0.239, *p* < 0.001).

## Discussion

To the best of our knowledge, this is the first study conducted using a unit-based observation approach, which separated the RCHs into different units according to the nature and intensity of care provision. Although there was no significant differences found among the different units, such information provided justification to inform future research. Furthermore, this study reported the changes of hand hygiene compliance over a period of 2–3 weeks. Surprisingly, there were no significant changes observed, thereby contradicting previous studies [23]. However, it was plausible that the hand hygiene performance was too low to explain the change. Floor effect was considered in this case.

Many researchers stated that hand hygiene is the most effective element in preventing infections. A systemic review of the impact of hand hygiene on risk infections in nursing homes revealed that the infection rate decreased when at least one hand hygiene-related intervention (e.g., availability of ABHR) was applied in the study [24]. However, the overall hand hygiene performance was still poor (15%) in the current study even though there was sufficient provision of ABHR. This result is similar with that of Smith et al. [25] and Ho et al. [26], who reported that the hand hygiene performance rate is 14.7% in two long-term care facilities and ranged from 19.5–27% in subsidized RCHs. Moreover, the availability of ABHR in common areas was better than in the bedroom areas among subsidized and private RCHs. Yet, there was no difference found in the hand hygiene performance between common areas and bedroom areas. This indicated that healthcare workers had no intention to perform hand hygiene even with the presence of ABHR, at least in this study. This contradicts past studies, which reported an increase in hand hygiene compliance with increased availability of ABHR [27, 28]. In fact, some studies showed that increase hand hygiene compliance not only increased ABHR but also staff education on the use of such material [25]. PCWs seldom used ABHR compared with other healthcare workers in the current study. Indeed, it was predicted that professionals would have higher hand hygiene compliance than PCWs and HWs due to differences in educational background. Studies on hand hygiene behavior always focused on professional staff in hospital settings. However, this may not reflect the same situation in the RCH setting in the current study, as many healthcare workers completed lower educational levels in RCHs (most staff were PCWs and HWs with secondary school education). Therefore, further assessment of hand hygiene behaviors among PCWs and HWs in the RCH setting is necessary.

In the present study, hand hygiene performance was the best after body fluid exposure risk (46% performed), after the aseptic procedure (25% performed), and after patient contact (25% performed) compared with before patient contact (2%) and after contact with the patients’ surroundings (3%). This result is consistent with previous research, which indicated a significantly higher hand hygiene performance after body fluid exposure, aseptic procedure, and after patient contact compared to before patient contact and after patient surrounding contact [29]. Furthermore, another study showed that the assistant healthcare workers had the worst hand hygiene compliance in each moment compared with nurses and doctors in Turkey [30]. The current study revealed a similar result in which PCWs (with similar job duties to assistant healthcare workers) had the worst performance in each moment compared with the professional staff. It is likely that healthcare workers think that the residents’ surroundings colonize with less microorganisms and lower risk of infection [29]. Moreover, in this study, the best performance in the use of gloves was the moment before coming into contact with the patients (38%) and before performing the aseptic technique (68%). It seemed that healthcare workers tend to protect themselves rather than protect others [31]. Therefore, evidence from studies should be shared to convince healthcare workers to practice hand hygiene effectively at each moment.

Meanwhile, wearing gloves diminished the transmission of organisms via the healthcare workers’ hand and protected them, whereas the improper use of gloves increased the risk of organism transmission [32]. The use of gloves, however, is not a substitute for hand hygiene. In fact, many studies have shown that hand hygiene is worse in case of using gloves. The current study also found that hand hygiene performance decreased with the increased use of gloves. PCWs had the most frequent contact with residents. Yet, they had the worst performance in hand hygiene and frequently improper use of gloves among the healthcare workers observed in the study. For example, they did not wash their hands after removing the gloves (missing = 98%) and did not change gloves between care procedures among residents (missing = 66.4%). Furthermore, they tended to perform hand hygiene only after completing specific successive tasks for all residents.

Respiratory tract infection (1.3%) is the most common type of infection in RCHs [7]. Respiratory protection is an effective preventive measure to prevent droplet transmission. In this study, most healthcare workers can correctly wear the surgical mask in case respiratory protection was required, yet 30% of healthcare workers failed to wear a surgical mask while performing patient care procedures. There was no observational study on wearing surgical mask in RCHs. The result is the same as that reported in a past study, which indicated that most healthcare workers wore a surgical mask correctly in the hospital setting [33].

Overall, the hand hygiene and respiratory protection performance scores showed no significant difference between common areas and bedroom areas in private and subsidized RCHs. However, the use of gloves had higher frequency in the bedroom areas than in common areas in the moments before performing an aseptic technique (i.e., wound dressing) and before coming into contact with bodily fluids (i.e., changing diaper). Respiratory protection, the use of gloves, and hand hygiene had just slightly better performance scores in subsidized than private RCHs. There was not enough significant difference observed in this study, although the manpower practice was much satisfactory in subsidized RCHs than in private ones. Some authors mentioned that ICP was performed better in subsidized RCHs with abundant resources [34]. However, the sample is not adequate to conduct a comparison with the current study.

Thus far, studies on ICP in Hong Kong RCHs by observational design without applying interventions have been limited. Wong et al. reported the first survey on the trends of ICP from 2005 to 2014, revealing that the overall trend of ICP was improved and that ICP in subsidized RCHs is better than in private RCHs due to the related factors (i.e., educational level of staff, resident-to-staff manpower ratio, and compliance with minimum statutory standards in private RCHs) [35]. However, Wong and colleagues only observed two healthcare workers (the infection control officer and one care worker) in each visit per year. Therefore, their results might not reflect the ICP among the majority of healthcare workers. In the current study, although comparisons between subsidized and private RCHs was limited, as the sampling size was not adequate, the results can still reflect the preliminary natural performance of ICP among healthcare workers, because continuous observations for 2–3 weeks were made, and the observations were carried out randomly during healthcare workers’ routine caring procedure in random instances.

The most observed ICP elements in this study were hand hygiene, use of gloves, and respiratory protection. However, other elements were not shown in this research, such as disinfecting used surfaces/equipment, handling of linen, handling of clinical waste, handling of sharp equipment, use of PPEs, and the likelihood of hand colonization. These elements were omitted from the observations, because these were not routinely performed and can only be observed by chance. In the future, we can obtain data from such practices by using a questionnaire if the procedure will be seldom observed. The generalizability of the study can also be improved by recruiting more participants in the sample of RCHs in future studies.

Meanwhile, healthcare workers had adequate levels of skills and knowledge on ICP [35]. However, ICP among healthcare workers was still poor in the current study. This implies that the adoption of ICP is always difficult to implement even if the staff are equipped with adequate knowledge. Compliance with ICP depends on a comprehensive theory behind [36]. PCWs are the main caregivers for residents in RCHs setting. Their workloads are much heavier than those of other types of healthcare workers in RCHs. Thus, future studies can give more focus on their ICP behaviors in this setting.

However, the findings of this study may not fully reflect the ICP practice during the COVID-19 epidemic. Based on the experiences learned when Hong Kong dealt with the severe acute respiratory syndrome (SARS) epidemic in 2003, many people reacted swiftly during the COVID-19 epidemic by wearing surgical masks in public areas and by following strict hand hygiene practices [37]. The residential care setting has been shown to be effective during the COVID-19 epidemic, in that no COVID-19 confirmed cases have been reported in RCHs or long-term care facilities at least in the first 6 months since the coronavirus first emerged in Wuhan in January [38]. RCHs are required by the Government of Hong Kong Special Administrative Region to promote infection control policies, including but not limited to, providing training on hand hygiene and the use of PPEs [39], thus resulting in higher ICP compliance in RCHs.

There are some limitations in the study that must be mentioned. First, whether the procedures were not part of the daily routine in some ICP episodes cannot be easily observed. Thus, the study failed to demonstrate the comprehensive ICP in RCHs. Second, the private RCHs refused to allow the researcher to observe the bed-bound elderly (18 residents) housed on the same floor. Hence, the results may not totally reflect the actual ICP compliance in private RCHs.

## Conclusions

Thus far, only a few studies on ICP in RCHs in Hong Kong have been conducted using the observational method over a period of time. To help address this gap, this study presents the preliminary phenomenon of ICP among healthcare workers in this setting. Overall ICP on hand hygiene, use of gloves, and respiratory hygiene was poor, thereby increasing the risk of HAI among healthcare workers and residents. In light of such findings, there should be continued monitoring and training among healthcare workers, particularly PCWs, who have the most frequent contact with residents in these healthcare service settings. Furthermore, appropriate infection prevention and control guidelines for this specific setting should be designed and implemented.

## Data Availability

The data and materials are available from the corresponding authors on reasonable request.

## Abbreviations

RCHs: Residential Care Homes
ICP: Infection control practice
HAI: Healthcare associated infection
HW: Health worker,
PCW: Personal care worker.
AHCP: Allied health care professionals
ABHR: Alcohol-based hand rub
